# Risk factors of malaria in children under the age of five years old in Uganda

**DOI:** 10.1186/s12936-016-1290-x

**Published:** 2016-04-27

**Authors:** Danielle Roberts, Glenda Matthews

**Affiliations:** School of Mathematics, Statistics and Computer Science, University of KwaZulu-Natal, Westville, Private Bag X 54001, 4000 Durban, South Africa

**Keywords:** Complex survey design, Generalized linear mixed model, Microscopy test, IRS, ITNs, LLINs

## Abstract

**Background:**

Malaria is the leading cause of morbidity in Uganda with 90–95 % of the population at risk and it contributing to approximately 13 % of under-five mortality. The aim of this study was to investigate the relationship between the malaria status of children under the age of 5 years old in Uganda and selected socio-economic, demographic and environmental factors, as well as to identify significant risk factors associated with malaria.

**Methods:**

This study made use of data collected from the 2014 Malaria Indicator Survey conducted in Uganda. Two test procedures for malaria in children under the age of 5 years old were carried out. Due to the complex survey design, a generalized linear mixed model was used to test for associations between several independent variables and the response variable, which was whether a child tested positive or negative for malaria according to the microscopy test.

**Results:**

The sample in this study was made up of 4939 children. Of those children, 974 tested positive for malaria, resulting in an observed malaria prevalence of 19.7 %. The socio-economic factors closely related to the risk of malaria were main floor material, main wall material and availability of electricity in the household. The event of indoor residual spraying (IRS) significantly reduced a child’s risk of malaria. An older child was associated with a higher risk of malaria, however their risk decreased with an increase in cluster altitude and an increase in their caregiver’s education level.

**Conclusion:**

Although there has been a significant increase in the use of mosquito nets since the previous Malaria Indicator Survey done in 2009, particularly in the use of insecticide-treated nets (ITNs) and long-lasting insecticidal nets (LLINs), these control measures alone may not be sufficient. IRS will be a key strategy in reaching the malaria goals set by the government of Uganda. Supplementing these control measures with education of appropriate and consistent use of ITNs and LLINs, as well as education of practicing safe living habits, such as reducing outdoor activities during peak biting hours of a mosquito, can go a long way in aiding the reduction of the burden of malaria in Uganda.

## Background

Malaria is considered to be one of the main global health problems, with it causing approximately 438,000 deaths in 2015 [1]. Ninety percent of these deaths occur in sub-Saharan Africa and 70 % are of children under the age of 5 years old. According to the World Health Organization (WHO), the number of deaths due to malaria in children under the age of 5 years has decreased significantly since 2000, and thus malaria is no longer considered the leading cause of death in children within this age group. However, malaria remains a major cause of morbidity in children in sub-Saharan Africa with 10 % of all deaths of children under the age of 5 years due to malaria. This is equivalent to one child in sub-Saharan Africa dying of malaria every 2 min [1]. Uganda, ranked third in the total number of malaria cases in sub-Saharan Africa, experiences weather conditions that often allow transmission to occur all year round with only a few areas that experience low or unstable transmission [2]. Climate affects both the parasite and the mosquito. Mosquitoes are unable to survive in low humidity and their breeding grounds are expanded by rainfall. *Plasmodium* parasites are affected by temperature where their development slows as the temperature drops and stops at high temperatures, which is the reason why parasites can be found in temperate areas [3]. Malaria is the leading cause of morbidity in Uganda with 90–95 % of the population at risk and it contributing to approximately 13 % of under-five mortality. [4]. Children under the age of 5 years are among the most vulnerable to malaria infection as they have not yet developed any immunity to the disease [5].

In Uganda, malaria control received little attention from the Ministry of Health before 1995, after which the Malaria Control Programme (MCP) was established in order to direct and guide the day to day implementation of the National Malaria Control Strategy. Today, the fight against malaria is part of the overall effort of the Government of Uganda, with the support of several partners, to improve health with an overall goal of reducing mortality due to malaria by 80 % of the 2010 levels and reducing morbidity due to malaria by 75 % of the 2010 levels by 2020 [6]. In these efforts, nationally representative cross-sectional surveys are carried out in the country in order to monitor and evaluate the progress of malaria control [7]. These include Demographic Health Surveys (DHS) which have been conducted every 5 years since 2001 and Malaria Indicator Surveys (MIS) conducted in 2009 and 2014/2015, in which two test procedures for malaria in children under the age of 5 years old were carried out. Both the DHS and MIS collect national and regional data from a representative sample of respondents and consist of a multi-stage sample design. A core part of the MCP is prevention of malaria, where insecticide-treated nets (ITNs) and long-lasting insecticidal nets (LLINs) continue to be distributed free of charge, and indoor residual spraying (IRS) is being carried out in a limited number of districts where malaria transmission is very high. Furthermore, in efforts to accelerate the reduction of child mortality, the Integrated Community Case Management (ICCM) was introduced in Uganda in 2013, which is part of the government’s Integrated Management of Childhood Illness (IMCI) strategy. These strategies involve Village Health teams (VHTs) offering curative treatments for malaria, diarrhea and pneumonia at community level, which assist in ensuring early diagnosis and treatment [8].

Although these control measures, together with those implemented by numerous non-profit organizations, have successfully reduced the number of malaria cases in Ugandan children over the past few years, there is still a notably high number of children under five dying from malaria daily [8]. Therefore, in order to apply successful implementations to substantially reduce the burden of malaria, there is a continuous need to understand the epidemiology and risk factors associated with the disease [9]. Although a large number of studies done worldwide have identified a wide variety of risk factors; socioeconomic, environmental, demographic, and others, associated with malaria infection [10–16], there is still a great need to identify the influence of these factors in a local context to allow a successful formulation of a national malaria-control strategy. There have been very few studies done in Uganda on malaria indicators and risk factors. These studies have also been specific to one or a few communities at a time [9, 17–20]. Most recent studies on malaria in Uganda have been hospital-based, investigating clinical malaria among young children and pregnant women [21–26].

The aim of this study was to use the data collected in the 2014–15 MIS carried out in Uganda to investigate the relationship between the malaria status of children under the age of 5 years old and selected socio-economic, demographic and environmental factors, as well as to identify significant risk factors associated with malaria.

## Methods

### Study area and sample design

The Republic of Uganda is a small, landlocked country located on the equator in East Africa. It shares borders with Sudan in the north, Kenya in the east, the Democratic Republic of Congo (DRC) in the west, and Tanzania and Rwanda in the south. The country has an area of 241,550 km^2^ of which approximately 41,743.2 km^2^ is taken up by open water and swamps, notably Lake Victoria in the south east and Lakes Albert and Edward in the west of the country [27]. This abundance of water bodies in the country provides great breeding grounds for the *Anopheles* mosquito, which is responsible for the transmission of malaria to humans. Uganda is administratively divided up into 111 districts and one capital city, Kampala.

Uganda experiences a favourable tropical climate, both for man and mosquitoes, due to its relatively high altitude with most of the Southwest lying between altitudes of 1300 and 1500 m above sea level. High mountain ranges above 1800 m are found in the border region in the Southwest with Rwanda and the DRC, the Rwenzori Mountains in the West and Mount Elgon in the East [2]. These areas are sometimes prone to epidemics, experiencing low or unstable malaria transmission.

Uganda experiences two rainy seasons per year, with heavy rains from March to May and light rains between September and December. The peak incidence of clinical malaria follows the peak of the rains with a delay of about 4–6 weeks, therefore most cases are seen between December and February, and May and July. However, rainfall decreases in the North region of the country, turning it into just one rainy season per year, thus the malaria season is more between May and November. Due to the regular rainfall, the Southwest and Central regions are rich in vegetation and fertile soil, resulting in high population densities. Thus, 87.9 % of the population is exposed to moderate to very high malaria transmission [26].

**Fig. 1:**
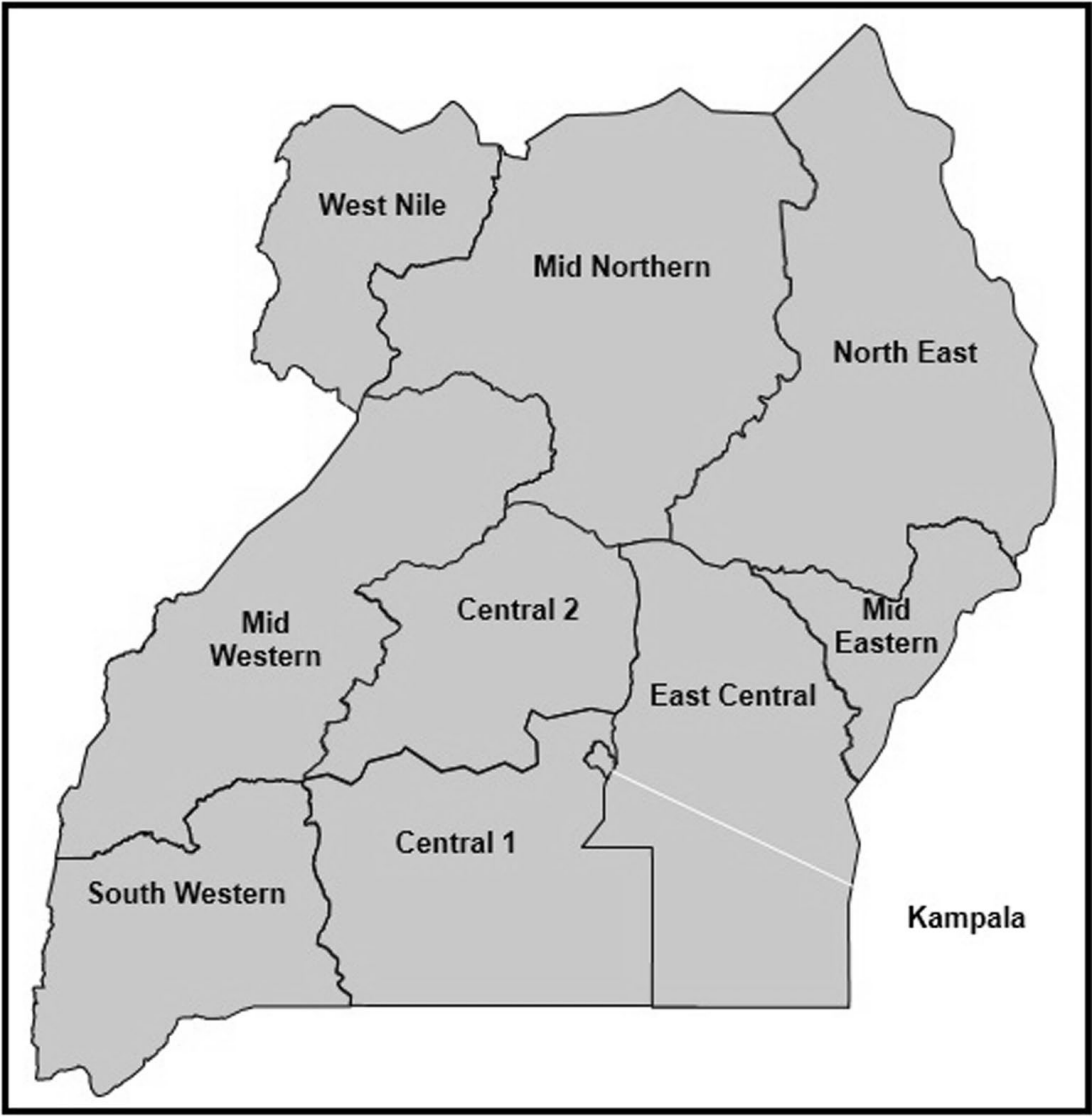
MIS 2014/15 sample regions in Uganda. Image taken from Uganda Bureau of Statistics (UBOS) and ICF's 2014/15 MIS methodology, household and respondent characteristics PowerPoint presentation

The 2014–15 MIS was carried out during December 2014 and January 2015 to correspond with peak malaria transmission. The sample was stratified into nine survey regions of the country, plus the capital city, Kampala, which, due to it being entirely an urban district, comprised a separate region. Each of the nine other regions consisted of 8–15 administrative districts of Uganda that shared similar languages and cultural characteristics. These 10 sampling regions of Uganda are shown in Fig. 1.

The sample was selected using a stratified two-stage cluster design. In the first stage, 20 sampling strata were created from which 210 clusters were selected by a probability-proportional-to-size. Thus, larger clusters (clusters containing more households) had a higher chance of being selected. A complete list of all households in the selected clusters was obtained 2 months before the survey took place, thus serving as the sampling frame from which the households would be selected in the second stage. The second stage involved selecting 28 households from each cluster by equal probability systematic sampling. This sampling method entailed selecting every $k^{\text {th}}$ household from an ordered list, where *k* is approximately equal to the cluster size divided by 28 (the number of households selected in each cluster).

### Uganda malaria indicator survey data

The selected households were visited and interviewed by trained staff. The Household Questionnaire collected basic information on the characteristics of each member and recent visitors of the household, including age and gender. The Household Questionnaire also collected information on characteristics of the household’s dwelling unit, such as source of water, type of toilet facilities, materials used for the floor, roof and walls of the house, ownership of various durable goods, and ownership and use of mosquito nets. The Woman’s Questionnaire was used to collect a range of information from all eligible women in the sample. With the consent of a parent or guardian in the household, all children between the ages of 0 and 59 months were tested for malaria and anaemia.

Two types of test procedures were used to determine the prevalence of malaria in the children; a rapid diagnostic test (RDT) and microscopy. The RDT consisted of testing a drop of blood using the SD Bioline Antigen rapid diagnostic test, which tests for the parasite *Plasmodium falciparum*, the most prevalent *Plasmodium* species in Uganda. The result of the test was available within 15 min. The second test procedure involved taking two blood smears; one thick and one thin. The thick smears were first examined by microscopy to determine *Plasmodium* infection, then the thin smears of all positive thick smears were examined to determine the species of *Plasmodium* parasite. Ethical approval was granted by ICF International’s institutional review board, the Makerere University School of Biomedical Sciences Higher Degrees Research and Ethics Committee (SBS-HDREC), and the Uganda National Council for Science and Technology (UNCST). Verbal informed consent was obtained from a child’s parent or guardian before tests were conducted.

### Response variable

Unlike the microscopy tests, RDTs are more readily available and do not require technicians with advanced skills and laboratories. However, the RDT detects the *Plasmodium falciparum*-specific protein (not the parasite itself), which can remain in the blood for several weeks after treatment. Therefore, this test can be less sensitive and may result in slightly higher rates of malaria. Therefore, for the purpose of this study, the prevalence of malaria in children under the age of 5 years was according to the microscopy test results. Thus, the response variable was binary, indicating whether a child tested positive or negative for malaria.

### Independent variables

The independent variables considered in this study comprised of a number of socio-economic, demographic and environmental factors. Such variables included gender and age of the child, number of members in the household, caregiver’s age, education level and knowledge of malaria, type of place of residence: rural or urban; cluster altitude and region of Uganda, main source of drinking water, type of toilet facilities, whether or not the household had electricity, a bicycle, television or a radio, main material of the floors, walls and roof of the household, incidence of IRS within the last 6 months prior to the survey, and ownership, treatment and use of mosquito nets in the household.

### The statistical model

Surveys carried out using sampling techniques such as multistage sampling, stratified random sampling, cluster sampling or sampling with unequal weights are often referred to as having complex survey designs [28]. Modeling of data obtained from these surveys must take into consideration the design of the study for the following reasons: (1) observations within the same cluster or household may be correlated and thus the assumption of independence in the data cannot be met. (2) A limited number of clusters are sampled thus leaving a significant portion of the population unsampled. This may result in certain characteristics not being represented in the study. (3) Sample units may be selected with unequal weights or probabilities. (4) Often surveys are subject to non-response. This may result in unmeasured characteristics which could lead to biased results.

Logistic regression, an extension of the generalized linear model (GLM), is commonly used to explore the relationship between a binary response variable and a set of explanatory variables. However, this method of analysis is not valid if the data come from complex survey designs, where the design of the study is such that the clusters included in the sample represent only a random sample from a population of clusters [29]. In the case of modelling the MIS data, it is also important to account for effects of clustering where children within the same cluster or household may be more alike compared to those from different clusters or households.

There are many methods of dealing with this design of the study. One such method is to include the effect of clustering in the model using a random effect. When a random effect is included in a GLM, the resulting model is referred to as a generalized linear mixed model (GLMM). This is referred to as a model-based method where, not only is there an interest on inferences concerning the effects of certain covariates on the response variable, but there is also an interest in estimating the proportion of variation in the response variable that is attributable to each of the multiple levels of sampling [29]. Therefore, in order to account for a possible correlation among the observations in this study, a GLMM was used.

## Results

### Study population

**Fig. 2:**
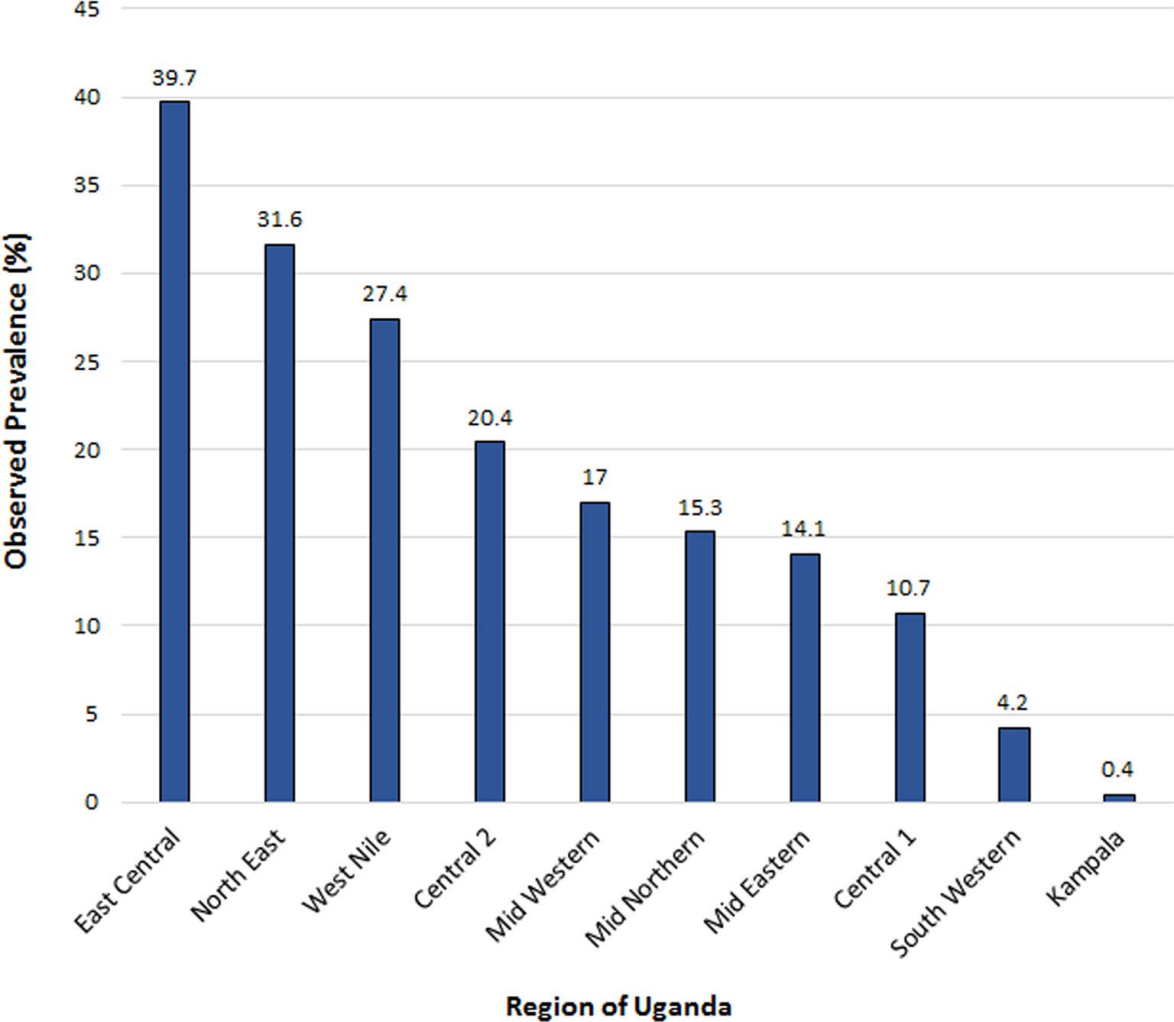
Observed prevalence of malaria within each sampled region of Uganda

The sample in this study was made up of 4939 children. Of those children, 974 tested positive for malaria, resulting in an observed malaria prevalence of 19.7 %. This was a substantial reduction compared to an observed malaria prevalence of 43.3 % in the 2009 MIS. Thus, indicating that control measures put in place between 2009 and 2014 have aided in reducing the burden of malaria in children under the age of 5 years. Figure 2 indicates that there was large variation in the prevalence of malaria across the different regions of Uganda. The East Central region, which experiences high malaria transmission and borders Lake Victoria, has the highest observed prevalence of 39.7 %. Some studies have suggested Lake Victoria is a fertile breeding ground for malaria vectors [30]. Kampala had the lowest prevalence with only 0.4 % of the children in the region testing positive.

The majority of the sampled households (79.3 %) were in clusters with altitudes ranging between 1000 and 1500 m, and 13.9 % were in clusters with altitudes higher than 1500 m where malaria transmission is lower. The total number of mosquito nets available in each sampled household was recorded. The maximum number of nets in a household was 7 with a median of 3 and a mean of 2.94 nets. A total of 96.3 % of households had at least one mosquito net and 76 % of the children in the sample slept under an LLIN the night before the survey took place. This was an increase from the 2009 MIS where the mean was 1 net per household and only 63.5 % of households had at least one net, with only 32.7 % of the sampled children having slept under an LLIN the night before the survey. Information about indoor residual spraying of the interior walls within the last 6 months prior to the survey was also collected. Only 9 % of the households had been sprayed at least once within the last 6 months.

Out of the sampled households, 12.9 % had electricity. The observed prevalence of malaria in children residing in households without electricity was 21.7 %, in contrast to only 5.2 % for those in households with electricity. Only 22.7 % of the households had a finished floor surface, this included tiled floors, cement, stones and bricks. Whereas 41.4 % of the households had only earth/sand as their main floor material, 34.9 % had a mixture of sand and dung, and 1 % had other floor materials unspecified in the survey questionnaire.

At 34.1 %, mud with poles or stone was the most common type of wall material in a household, followed by finished walls in 32.9 % of the households. These finished walls included cement walls, walls made with stones and cement, and burnt bricks with cement. Twenty percent (20 %) of the households had unburnt bricks with mud or plaster as the main wall material, 11.4 % used dirt to construct the walls in the household, and 0.9 % had other wall materials unspecified in the survey questionnaire. Unburnt bricks are dried with the help of heat received from the sun after the process of moulding, unlike burnt bricks which utilize the heat from a factory oven. Unburnt bricks are generally only used in the construction of simple temporary or cheap structures and should not be used at places exposed to heavy rains.

Out of the children tested for malaria, 48.7 % were male. The observed malaria prevalence for the male children was 19.8 % and that of the female children was 19.6 %. Thus, suggesting gender may not be a significant risk factor for malaria. The mean age of the children was 30.8 months (just over two and a half years old). The total number of members in each household ranged from 2 to 24, with a mean of 6.8 members. The majority of the children (49.1 %) had caregivers with only a primary school level of education, while 19.2 % had caregivers with no education, 13 % had caregivers with a secondary education and only 2.7 % had caregivers with an education higher than secondary school. Unfortunately, 16 % of the children had caregivers with an unspecified education level. These caregivers either did not know their highest education level or refused to state it. This group of caregivers may be associated with having a low level of education as it may be unlikely for a highly educated individual not to know their highest education level or to prefer not to specify it. A total of 89 % of the children in the sample had caregivers who knew mosquito bites can cause malaria, and 93.9 % had caregivers who knew there are ways of preventing malaria. However, only 20.2 % had caregivers who knew malaria can be avoided by sleeping under an insecticide treated net.

### GLMM applied to the MIS data

The analysis of the MIS data in this study was done using SAS version 9.3, furthermore, the procedure PROC GLIMMIX was used. In the case of a binary response variable, a logit link is used in the GLMM. In order to account for the heterogeneity between clusters in the MIS data, and thus a possible correlation among the observations, an intercept that varied at cluster level was included in the model, therefore resulting in a random intercept model. The model was fitted using the Laplace approximation as this method is likelihood based and therefore allows for the comparison of models using model selection criteria such as Akaike’s information criterion (AIC) and Bayesian information criterion (BIC). This method was also computationally less demanding. The need for a random intercept was assessed by testing if its corresponding covariance parameters equaled zero. The results of this test is shown in Table 1. Since the parameter under the null hypothesis fell on the boundary of the parameter space, the p value for the test was determined using a linear combination of central Chi-square probabilities. The result of this test indicates that the null hypothesis of the covariance parameter equal to zero was rejected (p < 0.0001), thus suggesting the random cluster effect was highly significant in the model.

**Table 1:** Test of covariance parameters

Label	DF	−2Log likelihood	Chi-square	p value
No G-side effects	1	4074.38	246.73	<0.0001

Before model selection of the fixed effects, the model was fitted with different covariance structures in order to determine which one best suited the data. The first structure fitted was SAS’s default, VC or variance components. Other structures fitted were AR (1) (autoregressive), CS (compound symmetry) and UN (unstructured). However, VC produced the lowest AIC value and was therefore selected. In order to obtain the final GLMM, a backward selection procedure was carried out where insignificant fixed effects, according to the p value of the fixed effects in the type III analysis (determined using the Wald F test), were removed from the model one at a time until only significant fixed effects were left. All p values less than 5 % were considered significant. To avoid possible confounding effects, all two-way and higher order interactions were explored, however none were found to be significant. The final model is shown in Table 2.

**Table 2:** Type III analysis of fixed effects for GLMM

Effect	Num DF	F value	p value
Age in months	1	108.17	<0.0001
Caregiver’s education level	4	4.67	0.0009
Region of Uganda	9	5.50	<0.0001
Cluster altitude in metres	1	9.45	0.0021
Household had electricity	1	5.01	0.0253
Main floor material	3	11.86	<0.0001
Main wall material	4	3.01	0.0173
Incidence of IRS within last 6 months	1	26.91	<0.0001

The age of the child in months, caregiver’s education level, region of Uganda, cluster altitude in metres, availability of electricity in the household, main floor material, main wall material and incidence of IRS within the last 6 months prior to the survey were all found to be significantly associated with a child’s malaria status. The Pearson Chi-square statistic over its degrees of freedom was 0.82, indicating there was no residual overdispersion. The variance component for the random cluster effect was estimated as 0.885 with a standard error of 0.150. This estimate is relatively far from zero, thus confirming again the need for this random effect in the model.

Table 3 presents the parameter estimates, odds ratios with their 95 % confidence intervals, and p values for the fixed effects of the final model. These results revealed that the odds of malaria increased as the age of a child increased (OR 1.028, 95 % CI 1.023–1.033). However, the odds of malaria for a child decreased with an increase in cluster altitude (OR 0.999, 95 % CI 0.998–0.999), specifically, the odds decreased by approximately 10 % as the cluster altitude increased by 100 m. Compared to children who had caregivers with a secondary education, those with caregivers that did not specify their education level were most at risk for malaria (OR 1.966, 95 % CI 1.349–2.866). These odds were very similar to those with caregivers who had no education (OR 1.961, 95 % CI 1.345–2.858), thus confirming the idea that the caregivers with the unspecified education level may have had a low level of education or none at all. Followed by these two groups were children with caregivers who had only a primary education (OR 1.589, 95 % CI 1.126–2.242). Even though the odds of malaria for children who had caregivers with a higher education were only 0.402 times that for children who had caregivers with a secondary education, there was no significant difference between them (p = 0.2463).

**Table 3:** Estimates and odds ratios (OR) with 95 % confidence intervals

Parameter	Estimate	OR	95 % CI	p value
Intercept	−6.386			<0.0001
Age in months	0.028	1.028	1.023–1.033	<0.0001
*Caregiver’s education level (ref. = secondary)*				
No education	0.673	1.961	1.345–2.858	0.0005
Primary	0.463	1.589	1.126–2.242	0.0083
Higher	−0.912	0.402	0.086–1.877	0.2463
Unspecified	0.676	1.966	1.349–2.866	0.0004
*Region of Uganda (ref. = South Western)*				
Central 1	1.176	3.241	1.196–8.782	0.0208
Central 2	1.413	4.107	1.579–10.686	0.0038
East Central	2.476	11.897	4.579–30.912	<0.0001
Kampala	−1.258	0.284	0.037–2.195	0.2277
Mid Eastern	0.926	2.525	1.031–6.180	0.0426
Mid Northern	1.697	5.459	2.038–14.626	0.0007
Mid Western	1.040	2.830	1.137–7.045	0.0253
North East	2.104	8.199	3.521–19.089	<0.0001
West Nile	1.667	5.294	1.968–14.238	0.001
Cluster altitude in metres	−0.001	0.999	0.998–0.999	0.0021
Household had electricity (ref. = yes)	0.563	1.756	1.072–2.877	0.0253
*Main floor material (ref. = finished surface)*				
Earth/sand	0.974	2.650	1.870–3.754	<0.0001
Sand and dung	0.591	1.806	1.231–2.649	0.0025
Other	−0.135	0.873	0.221–3.457	0.8471
*Main wall material (ref. = unburnt bricks)*				
Dirt	0.388	1.474	0.995–2.184	0.0532
Finished walls	0.476	1.609	1.157–2.239	0.0047
Mud and poles/stone	0.472	1.603	1.141–2.251	0.0065
Other	−0.227	0.797	0.323–1.966	0.6218
Incidence of IRS within last 6 months (ref. = yes)	2.035	7.649	3.545–16.501	<0.0001

Compared to children who resided in the South Western region of Uganda, which had the second lowest observed prevalence of malaria, children who resided in all the other regions of Uganda, except Kampala, were more at risk with the odds ratios ranging from 2.525 in the Mid Eastern region to 11.897 in the East Central region (which had the highest observed prevalence). While the odds of malaria for children in Kampala was only 0.284 times that of children in the South Western region, there was no significant difference between the two regions (p = 0.2277).

Children in households without electricity were more than one and a half times more likely to have malaria than those in households with electricity (OR 1.756, 95 % CI 1.072–2.877). Children residing in households with just the earth/sand as the main floor material were most at risk for malaria compared to those in households with a finished floor surface (OR 2.650, 95 % CI 1.870–3.754), followed by those in households with sand and dung on the floors (OR 1.806, 95 % CI 1.231–2.649). Compared to children in households with unburnt bricks as the main wall material, finished walls, walls constructed from dirt, and walls constructed with mud and poles or stones were associated with a higher risk of malaria, with the odds of malaria ranging from 1.474 to 1.609. Children residing in households with finished wall surfaces had the highest odds of malaria compared to those in households with unburnt bricks as the main wall material (OR 1.609, 95 % CI 1.157–2.239). There was no significant difference between the unspecified wall material and unburnt bricks (p = 0.6218). The occurrence of IRS in a household within the last 6 months prior to the survey significantly reduced the risk of malaria with the odds being 7.649 times higher for children residing in households that had not been sprayed during this period (95 % CI 3.545–16.501).

By adding the cluster effect into the model as a random effect, the heterogeneity between clusters is accounted for, however there may be an extra source of variation between households within clusters. It may be the case that children within the same household are more homogeneous than those from different households within the same cluster, and with some households having had up to 7 children tested for malaria and a mean of 1.6 children tested per household, it may be necessary to account for possible correlations that may exist within the households. Therefore, households nested within clusters were further added as a random effect. In fitting the full GLMM with this additional random effect, the test of covariance parameters once again produced a significant result with a p value <0.0001. However, the final model produced very similar results to that with only the cluster effect, therefore similar conclusions can be drawn.

## Discussion

The aim of this study was to investigate the relationship between the malaria status of children under the age of 5 years old and selected socio-economic, demographic and environmental factors, as well as to identify significant risk factors associated with malaria. Based on the results of this study, the socio-economic factors closely related to the risk of malaria were main floor material, main wall material and availability of electricity in the household. While electricity in a household could be related to one’s socio-economic status, it could also contribute to the individual’s way of life, where those in households with no electricity may be required to go outside more often and therefore may be more susceptible to mosquito bites, and thus malaria. None of the availability of household items such as a radio, television and bicycle, were found to be significantly associated with malaria. However, many of these variables could be explained by the inclusion of availability of electricity in the model. Poor/unimproved flooring quality was associated with a higher risk of malaria compared to a finished surface of tiles, cement or stones and bricks. However, a finished wall surface of cement, stones with cement, or burnt bricks with cement, was associated with an increased risk of malaria compared to unburnt bricks which are generally deemed as poor quality. This surprising result is contradictory to several other studies which have shown that poor and unimproved housing quality, specifically that of walls, is associated with a higher risk of malaria [11, 13, 31–33]. These studies note how poor housing quality facilitate mosquito entry and thus transmission of malaria. While a finished wall surface may not be associated with a higher risk of malaria due to its ability to allow mosquitoes entry into a household (as it is expected that this improved wall material is more secure than mud or dirt walls), it is possible that households with these finished wall surfaces may have additional characteristics aiding in the increased risk of malaria. One such characteristic could be the number or type of windows in the household. More secure wall finishes such as cement and burnt bricks with cement may allow for the construction of more windows, thus creating more entry points for the mosquito if these windows are not closed correctly or during peak biting times. Okebe et al. [32] and Bradley et al. [34] showed in their studies that there was strong evidence of an association between type of windows and malaria prevalence. However, no information about the number or type of windows in the households was collected in this MIS and thus cannot be explored. The quality of these finished wall surfaces may also be a contributing factor that may need to be explored.

The use of a mosquito bednet, whether or not the bednet was treated, the number of mosquito nets in the household and whether or not the child slept under an LLIN the night before the survey were insignificantly associated with a child’s malaria status. These results are in agreement with those found by Gahutu et al. [12] and Okebe et al. [32] however they conflict the results found by Ayele et al. [13], Baragatti et al. [35], Winskill et al. [36] and Wotodjo et al. [37]. This observed lack of association could possibly be attributed to an inconsistent or inappropriate use of the nets or perhaps a child was exposed to mosquito bites during other times of the day or evening when the net was not in use. However, incidence of indoor residual spraying within the last 6 months was significantly associated with a child’s malaria status where the risk of malaria was substantially reduced in the event of IRS.

The region of Uganda that a child resided in was shown to be significantly associated with the risk of malaria, with children residing in the East Central and North West regions having the highest risk compared to those in the South Western region. Surprisingly, the type of place of residence (rural versus urban) had an insignificant association with the risk of malaria, contrary to the study done by Liu et al. [38]. This could be due to the majority of the country being classified as rural with 84 % of the people of Uganda considered as part of the rural population [39]. This rural population is still found in areas considered as urban. The type of place of residence could also be explained by the inclusion of the region of Uganda in the model.

Similar to studies carried out by Pullan et al. [9], Gahutu et al. [12] and Krefis et al. [40] an older child was associated with a higher risk of malaria. However, their risk decreased with an increase in cluster altitude. Gender was found to be insignificant, with the distribution of malaria prevalence being almost the same for both males and females, which is consistent with the findings of other studies [13, 35, 41]. The education level of a individual, particularly that of a child’s caregiver, has been shown to be an important risk factor of malaria, in this study and others [42, 43]. It is assumed that more educated individuals have a better understanding of health related issues. In regards to malaria, compared to children who had caregivers with a secondary education, those with caregivers who had an unspecified level of education or no education were most at risk. However, a caregiver’s knowledge of malaria was not found to be associated with a child’s risk of malaria.

The limitations of this study include not being able to make meaningful inferences about the category ‘other’ in the two variables main floor material and main wall material, as well as the ‘unspecified’ education level of caregivers. Another limitation is that the Malaria Indicator Survey was a cross-sectional study, therefore whether a child who tested negative for malaria had previously been infected was not taken into consideration. This design of study also hindered establishing a temporal sequence of association between significant risk factors and malaria. However, the study has its strengths. The complex design of the study was accounted for by including the effect of the clusters in the model as a random effect. The test of covariance parameters confirmed that accounting for this cluster effect was necessary in order to make meaningful and accurate statistical inferences. The use of a multivariate GLMM allowed an assessment of the association between the prevalence of malaria and a potential risk factor, while adjusting for other variables. In this study, several important socio-economic, demographic and environmental risk factors for malaria were identified using a population-based survey.

## Conclusion

While some of the results of this study agreed with those of other studies, some new findings came to light which require further exploration. Particularly with respect to households with finished wall surfaces of cement, stones with cement, or burnt bricks with cement. The large up-scaled efforts of malaria control in Uganda can be seen since the last MIS in 2009, however, Uganda still has a long way to go before reaching its targeted levels of morbidity and mortality due to malaria in 2020. Although there has been a significant increase in the use of mosquito nets, particularly ITNs and LLINs, these control measures alone may not be sufficient. IRS will be a key strategy in reaching these goals. Supplementing these control measures with education of appropriate and consistent use of ITNs and LLINs, as well as education of practicing safe living habits, such as reducing outdoor activities during peak biting hours of a mosquito, can go a long way in aiding the reduction of the burden of malaria in Uganda. The extent of the under-development of the country also presents a great challenge in the efforts of malaria reduction, especially as approximately a quarter of the people live below the national rural poverty line [44]. As resources for malaria control in Uganda are limited, and the different regions of the country have been shown to be unequally at risk, it is of great importance to identify the geographical areas that are most at risk through updated malaria risk maps. Risk maps, created through spatial modelling, have been recognized as an important tool for malaria control where they can effectively guide the allocation of the limited resources and interventions.
